# Endoscopic retrograde cholangiopancreatography in a patient with complete situs inversus viscerum: A case report and literature review

**DOI:** 10.1002/deo2.17

**Published:** 2021-05-27

**Authors:** James Emmanuel, Nagaraj Sriram, Raman Muthukaruppan

**Affiliations:** ^1^ Department of Gastroenterology and Hepatology Queen Elizabeth Hospital Kota Kinabalu Sabah Malaysia

**Keywords:** choledocholithiasis, endoscopic retrograde cholangiopancreatography, situs inversus

## Abstract

Complete situs inversus viscerum (SIV) is a rare congenital condition, defined by a left‐right transposition of all viscera with dextroposition of the heart. In patients with SIV that requires endoscopic intervention, namely endoscopic retrograde cholangiopancreatography (ERCP), the left‐right coordination can be technically demanding even with skilled endoscopist. We report a case of a patient with underlying SIV who presented with septic shock secondary to ascending cholangitis compounded with a malaria infection. Despite the ascertainment of a relatively large Common Bile Duct (CBD) stone, ERCP and stenting were pursued as an initial treatment modality in view of the clinical presentation of cholangitis and COVID‐19‐related delays in surgical intervention at our center. This case is unique as the patient was maintained in a supine position throughout the procedure. The patient underwent a successful ERCP procedure followed by a CBD Exploration and cholecystectomy 2 weeks later. A key factor that contributed to the success of this procedure was the combined utilization of a rotatable sphincterotome and extractor balloon which assisted with cannulation and shortening manoeuvre of the duodenoscope to facilitate biliary stenting.

## INTRODUCTION

Complete situs inversus viscerum (SIV) is a rare congenital condition, defined by a left‐right transposition of all viscera with dextroposition of the heart.^1^ The anatomical mirroring of visceral structures in patients with this anomaly is rarely discovered until a medical or surgical situation that warrants imaging presents itself. In patients with SIV that do present with a condition that requires endoscopic intervention namely endoscopic retrograde cholangiopancreatography (ERCP), the left ‐right coordination can be technically demanding even with skilled endoscopist.^1^, ^2^ Previously reported cases of ERCP in patients with SIV have laid major emphasis on the positioning of patient‐endoscopist, to perform a successful procedure.^1^, ^3^ However, our case is unique as the entire procedure was carried out with the patient in supine position and in addition, we utilized a rotatable sphincterotome to engage the papilla with ease. A review of related reports has been included in the literature.

## CASE REPORT

**FIGURE 1:**
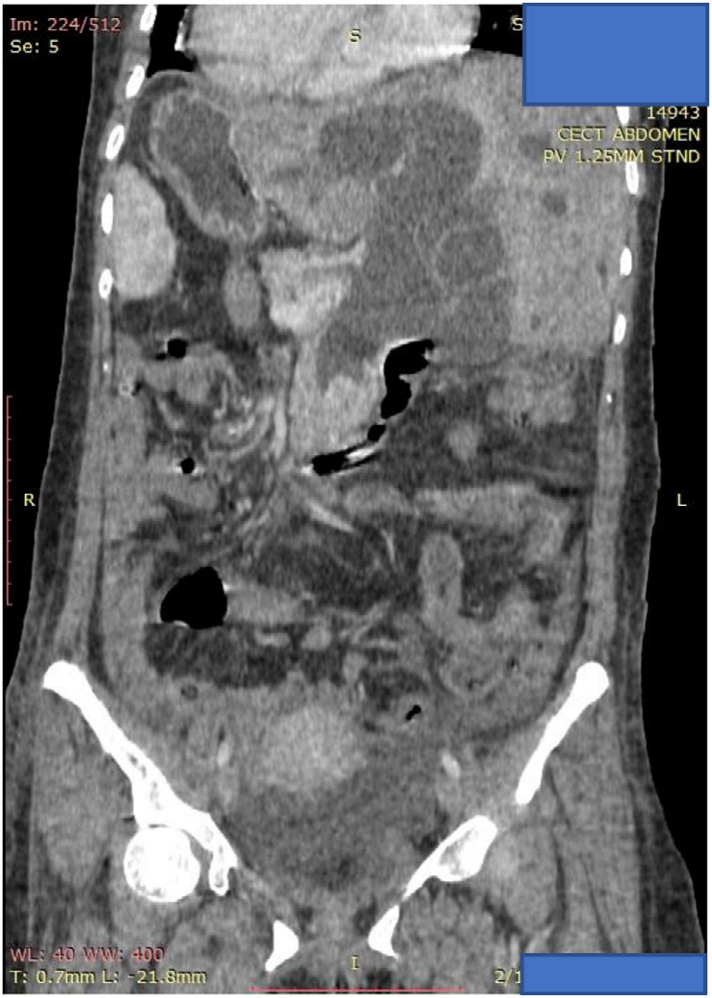
CT imaging revealed choledocholithiasis with upstream dilatation of the biliary tree

**FIGURE 2:**
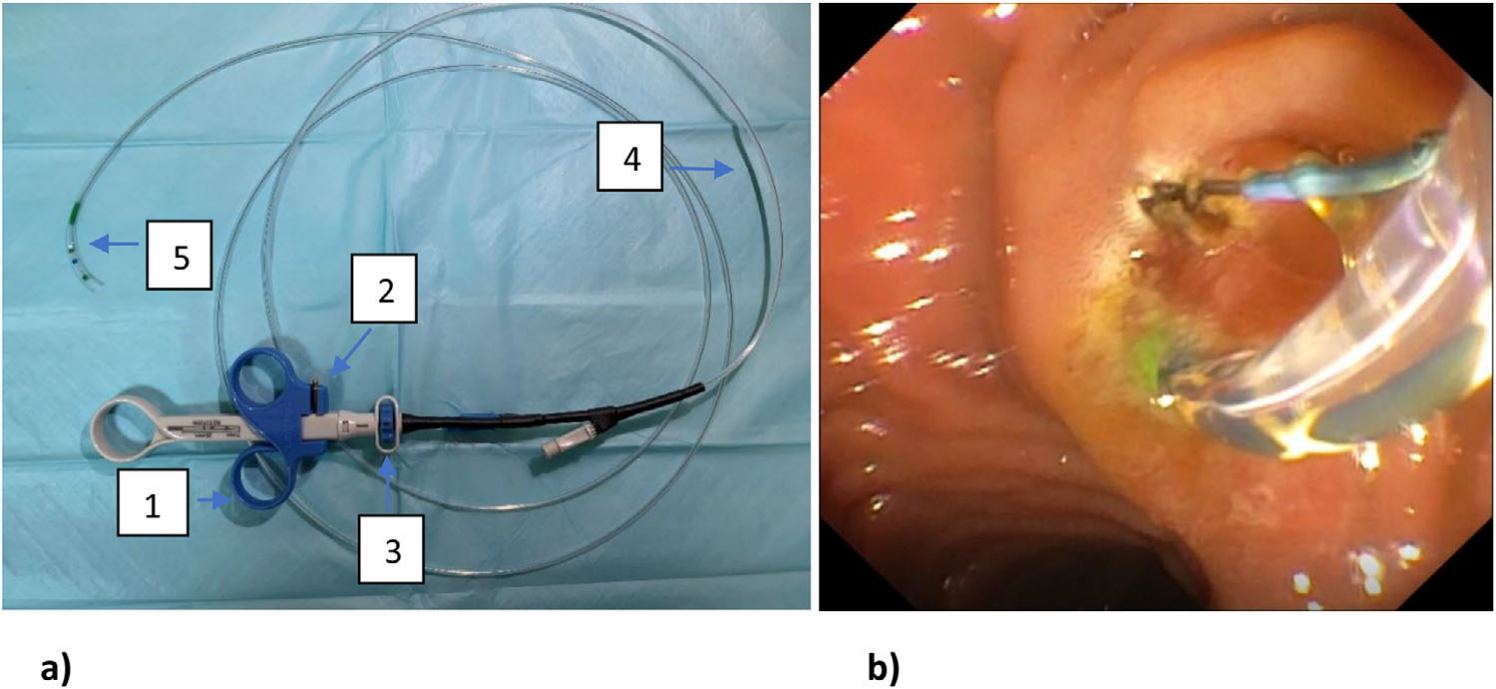
(a) Leo Med triple lumen sphincterotome. (b) Endoscopic sphincterotomy using the rotatable sphincterotome

**FIGURE 3:**
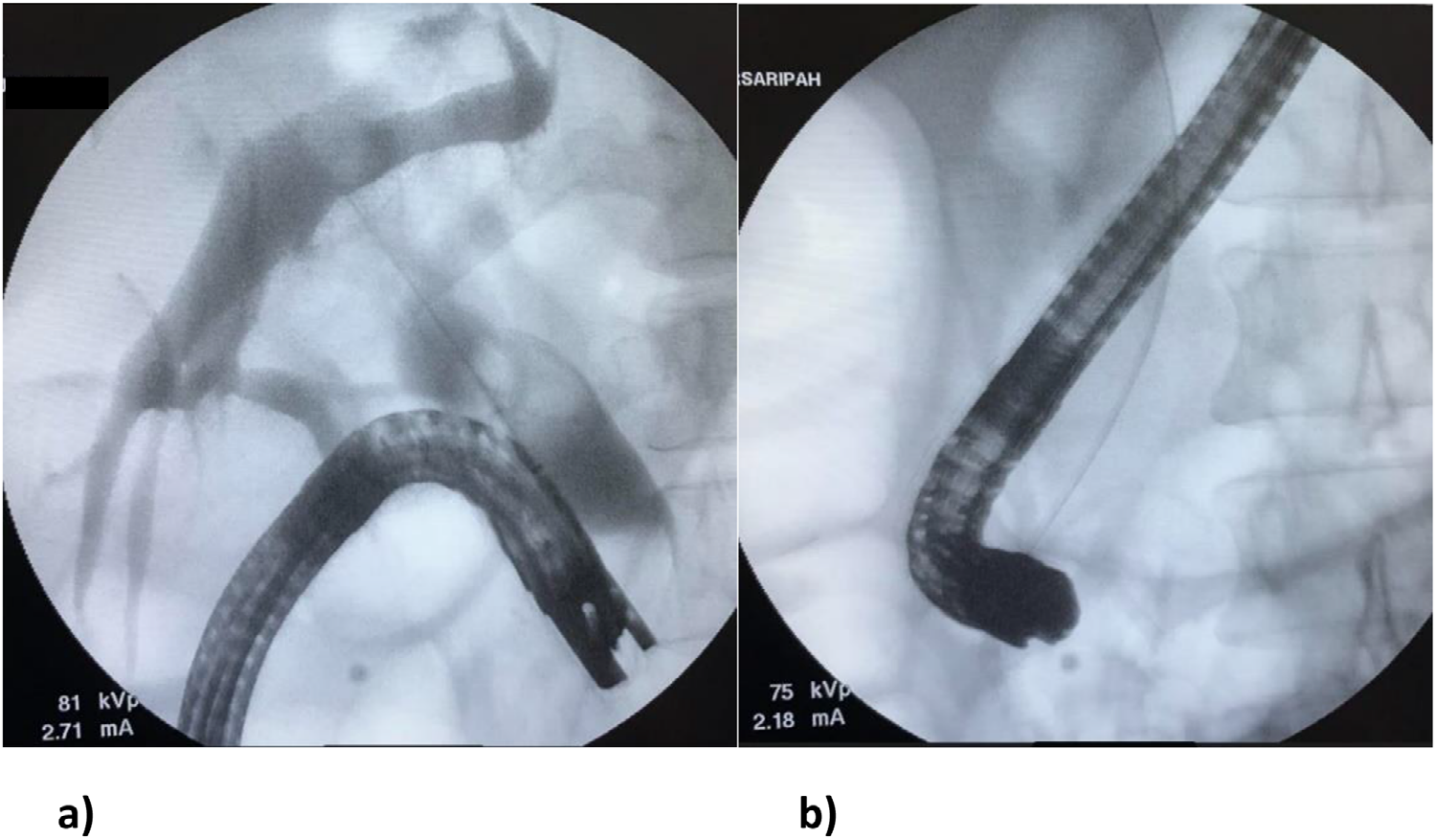
(a) Extractor balloon used to perform the shortening manoeuvre. (b) Duodenoscope position after shortening manoeuvre

**FIGURE 4:**
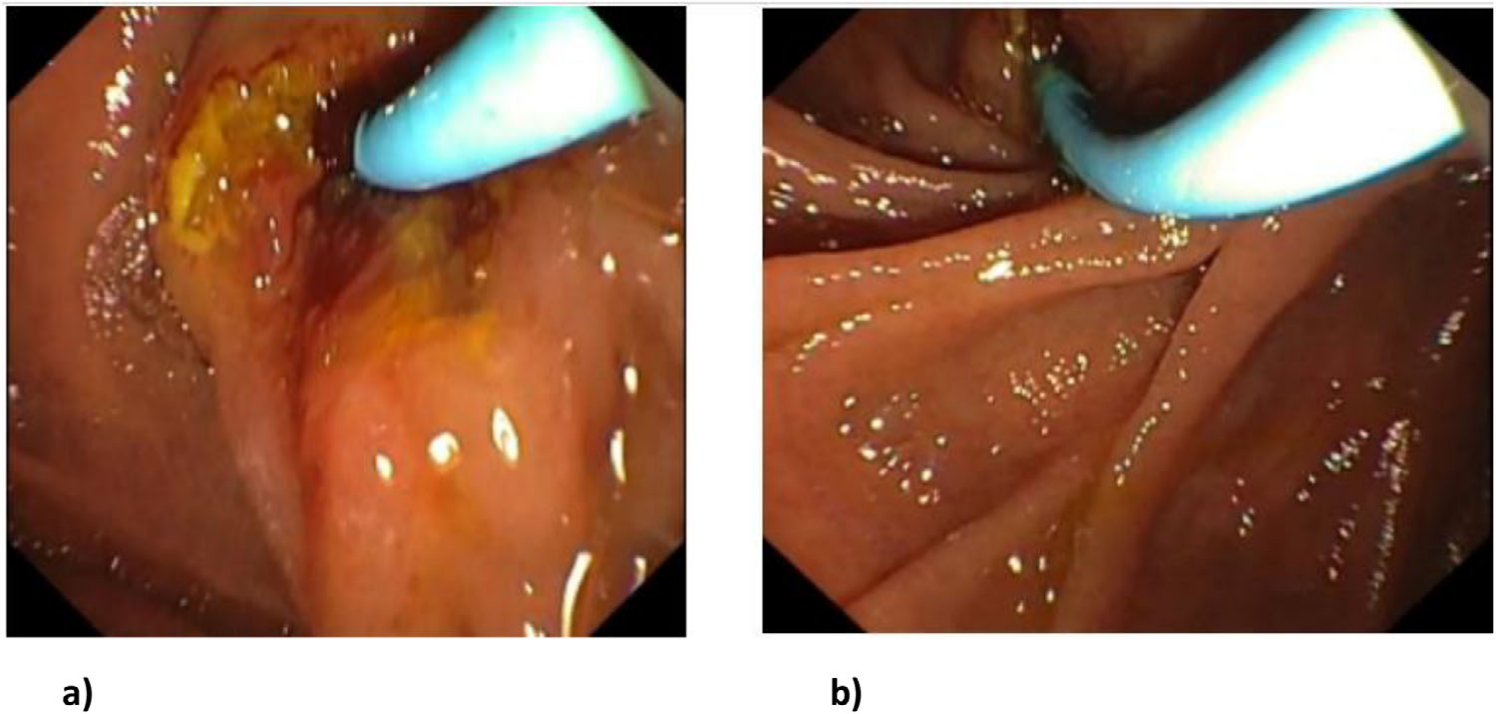
(a) Papilla at 2 o'clock position. (b) Papilla restored to the 11 o'clock position after the shortening technique

A 42‐year‐old female with no prior medical illness presented with fever, epigastric pain, and vomiting of 3 days' duration. On assessment in the Emergency Department, she appeared septic with a blood pressure of 70 of 46 mm Hg and a pulse of 98/min. Temperature was 37.5°C. Further examination revealed that she was icteric. She had crackles on both lung bases with the apex beat appreciated at the right 5th intercostal space. Blood parameters were notable for deranged Liver Function Test with total bilirubin, 5.4 mg/dL; aspartate aminotransferase (AST), 481 U/L; and alanine aminotransferase (ALT), 333 U/L. The Blood Film for Malaria Parasite which was positive for Plasmodium Falciparum added to the complexity of the case. Radiographic imaging of the chest was consistent with situs inversus. She was initiated on antibiotics, antimalarials, and vasopressors. CT imaging revealed choledocholithiasis with upstream dilatation of the biliary tree (Figure 1). There were no associated anatomical abnormalities of the CBD. The size of the CBD stone (2.2 × 1.8 cm) depicted on the CT had raised concerns on the amenability of an endoscopic intervention in achieving stone clearance. However, during the multidisciplinary meeting, a decision was reached to proceed with ERCP and biliary decompression as a temporizing measure in view of the anticipated delay in surgery due to ongoing COVID‐19 pandemic. Patient was placed in supine position with the endoscopist on the left side. The supine position was preferred by the anaesthetist for better control of the airway in the event of a desaturation episode. Following administration of sedation with propofol (100 mg), ERCP was performed with a side‐viewing endoscope (TJF‐Q180V J; Olympus, Tokyo, Japan). The endoscope was advanced into the duodenum and was rotated 180° clockwise with some degree of torsion. The papillary orifice was noted in the 2 o'clock direction, with the scope in long position. Owing to the location of the papilla, a rotatable sphincterotome (Leo Med triple lumen sphincterotome RST0725N, Leo Medical Co., Ltd, Changzhou City, Jiangsu Province, China) (Figure 2a) was employed to achieve deep biliary cannulation. Cholangiogram revealed a large mid CBD stone following which, a wire‐led sphincterotomy was performed (Figure 2b). Fluoroscopy image was mirrored to make cholangiogram interpretation more familiar. We then advanced a stone extraction catheter (Leo Med stone extraction catheter QS‐2A00, Leo Medical Co., Ltd, Changzhou City, Jiangsu Province, China) into the CBD and after inflating it we used the balloon as an anchor to perform the shortening manoeuvre of the duodenoscope (Figure 3a). This was essential as we had anticipated technical difficulty in extracting the stone owing to the axis between the 2 o'clock position of the ampulla and the duodenoscope. This manoeuvre was done by maintaining traction on the duodenoscope while performing a 180˚ clockwise torque. With this technique, we attained a short position of the endoscope (Figure 3b) and restored the papilla to the conventional 11 o'clock position (Figure 4). Nevertheless, attempts for CBD stone removal using an extractor balloon catheter failed in view of the size of the stone as well as the angulation of the duodenoscope. We proceeded with stenting using a straight plastic stent to decompress the biliary system. The procedure took approximately 35 min, and there were no episodes of desaturation throughout the procedure. Our patient had a favorable recovery following the procedure, with improvement of both clinical and biochemical parameters. She underwent Common Bile Duct Exploration (CBDE) and cholecystectomy 2 weeks following the ERCP procedure and was discharged well.

## DISCUSSION

There have been major advances in the field of interventional endoscopy; however these refinements have not in the most part, addressed patients with anatomic anomalies.^3^ Patients with underlying SIV pose significant challenges to the endoscopists performing an ERCP procedure. Current available literature on ERCPs performed in patients with SIV has been restricted to case reports due to its rarity. Our patient with underlying SIV presented with septic shock secondary to ascending cholangitis compounded with a malaria infection. Despite the ascertainment of a relatively large CBD stone in a case of SIV, ERCP and stenting were pursued as an initial treatment modality in view of the clinical presentation of cholangitis and COVID‐19‐related delays in surgical intervention at our center. There has been considerable heterogeneity in ERCP techniques as well as positioning of patient and endoscopist in previously reported cases of patients with SIV.^1^, ^2^, ^3^, ^4^, ^5^ Majority of the previously published cases had reported patients being placed in the prone position **(Previous reports of ERCP procedures done for cases of choledocholithiasis in patients with underlying SIV are summarized in** Table 1). In our case, factors such as the position of the inbuilt C‐ arm did not permit flexibility to the position of the endoscopist, thus necessitating a left sided approach. Interestingly, this is only the second reported case where a patient was maintained in a supine position throughout the procedure. Rocha et al had previously reported a successful ERCP procedure with a patient in supine position.^5^ Their inclination for this approach was with the rationalization that a supine position in a patient with SIV would render a similar disposition of organs when compared to a patient with normal anatomy placed in the conventional prone position. In our case this position was adopted due to risk of deterioration during the procedure which would demand immediate measures to secure the airway and institute resuscitation protocols. The use of a rotatable sphincterotome to accommodate for the anatomical anomaly as demonstrated in this case was a key determinant in achieving successful biliary cannulation and paving the way for us to shorten the duodenoscope thus restoring the papilla to its ideal position. With a rotational capacity of −45° to +45° to the initial direction, the advantage of this accessory is that it preferentially guides the directional axis of the catheter when cannulating a papilla which is not at the standard 11–1 o'clock position or when confronted with an intra‐diverticular papilla.^6^ Ideally we would have attempted to remove the stone by performing endoscopic large balloon dilation and employing the use of a mechanical lithotripter, however considering that the patient was clinically unstable and we had failed initial attempts of stone removal by conventional balloon trawling, we therefore opted to proceed with biliary stenting instead. The patient underwent a successful ERCP procedure followed by a CBDE and cholecystectomy 2 weeks later.

**TABLE 1:** Previously reported cases of ERCP for choledocholithiasis in patients with underlying SIV

Study	Year	Patient's position	Endoscopist position	Technique	Complications
Venu et al^4^	1985	Right lateral	Right side of the table	Patient's position was altered several times to facilitate biliary cannulation.	No
De la Serna‐Higuera et al^7^	2010	Prone	Right side of the table	Endoscope was turned 180° clockwise in the stomach followed by the use of a rotating sphincterotome.	No
García‐Fernández et al^8^	2010	Right lateral	Right side of the table	“Mirror image” technique. Endoscopic manoeuvres were performed inversely as per normal procedures	No
Lee et al^9^	2010	Prone	Right side of the table.	Endoscope was rotated 180° to the right in the stomach. Large‐balloon dilatation was performed after a limited sphincterotomy	No
Mathur et al^10^	2012	Prone	Right side of the table.	Endoscope was rotated 180° in the 2nd part of duodenum.	No
Yi Hu et al^1^	2015	Supine	Left side of the table	Patient was repositioned to a prone position once the endoscope was in the 2nd part of the duodenum. Endoscope was rotated 180° clockwise in the duodenum with some torsion followed by shortening.	No
Lee et al^2^ **(Two techniques)**	2017	Prone	Right side of the table.	**1st**: Endoscope was rotated 180° counterclockwise in the stomach and in the duodenum; the endoscope was again shortened using a 180° counterclockwise rotation.	No
				**2nd**: Endoscope was guided along the lesser curvature, while slowly rotating clockwise.	
Ihab I. El Hajj	2017	Prone	Right side of the table.	Endoscope was turned 180˚ to the right in the stomach and, under fluoroscopic guidance, D2 was reached. Another 180˚ torsion to the right was necessary in D2 to retrieve the scope in the short position.	No
Uma Devi et al^3^	2019	Left lateral position	Left side of the table.	By turning to the left, scope is advanced into the duodenum. Papilla made enface by counter clockwise rotation (left 90°) and withdrawing the scope.	No
Rocha et al^5^	2019	Supine	Left side of the table	Papilla was in the right upper quadrant, and bile duct cannulation was made toward the “1 o'clock” direction. After sphincterotomy, several infracentimetric stones were removed with a Dormia basket.	No
Current study	2020	Supine	Left side of the table.	Endoscope was rotated 180° clockwise in the duodenum with some torsion. A rotatable sphincterotome was used to facilitate biliary cannulation. Extractor balloon was used as an anchor to perform the shortening manoeuvre of the endoscope.	No

## CONCLUSION

We report a case of SIV presenting with ascending cholangitis, in whom ERCP was successfully performed in the supine position. A key factor that contributed to the success of this procedure was the contemporary utilization of a rotatable sphincterotome and extractor balloon which assisted with cannulation and shortening manoeuvre of the duodenoscope to facilitate biliary stenting.

## CONFLICT OF INTEREST

The authors declare no conflict of interests for this article.

## PATIENT CONSENT

The patient had given verbal consent for publication of details of the case.

## ETHICS STATEMENT

This study was conducted in compliance with ethical principles outlined in the Declaration of Helsinki and Malaysian Good Clinical Practice Guideline.

## FUNDING INFORMATION

None.
